# Impact of varying range of maternal oxygenation targets on fetal oxygenation and fetoplacental circulation in an ovine model of pregnancy

**DOI:** 10.1038/s41390-026-04858-z

**Published:** 2026-03-18

**Authors:** Nithi Fernandes, Satyan Lakshminrusimha, Praveen Chandrasekharan, Munmun Rawat, Sylvia Gugino, Justin Helman, Michelle J. Lim

**Affiliations:** 1 Department of Pediatrics, Division of Neonatology, Children’s Hospital of Michigan, Central Michigan University, Mount Pleasant, Ml, USA.; 2 Department of Pediatrics, Division of Neonatology, UC Davis Children’s Hospital, UC Davis School of Medicine, Sacramento, CA, USA.; 3 Department of Pediatrics, Division of Neonatal-Perinatal Medicine, Oishei Children’s Hospital of Buffalo, University at Buffalo, Buffalo, NY, USA.; 4 Department of Pediatrics, Division of Critical Care, UC Davis Children’s Hospital, UC Davis School of Medicine, Sacramento, CA, USA.

## Abstract

**BACKGROUND:**

Optimal oxygen levels in pregnant mothers undergoing mechanical ventilation are not known. This study examined the effects of four maternal oxygenation ranges on fetal hemodynamics and oxygenation in late-preterm lambs.

**METHODS:**

Thirty-seven ewes were intubated, sedated, and surgically catheterized, while exposed to varying FiO_2_ concentrations (0.1–1.0). Fetal lambs were partially exteriorized via hysterotomy for serial carotid blood gas and flow measurements. Maternal oxygenation was categorized as hyperoxia (PaO_2_ > 150 mmHg), normal (81–150 mmHg), ARDSnet target (55–80 mmHg), and hypoxemia (<55 mmHg).

**RESULTS:**

Fetal carotid oxygen content was comparable between hyperoxia and normal oxygenation with stable cerebral oxygen delivery (DO_2_: 2.0 ± 1.2 vs. 2.1 ± 1.7 mL/kg/min). In contrast, PaO_2_ 55–80 mmHg and <55 mmHg were associated with significantly reduced fetal oxygen content (5.2 ± 3.7 and 4.0 ± 3.1 mL/dL, respectively) versus >150 mmHg, though only <55 mmHg differed significantly from 81–150 mmHg. Cerebral DO_2_ trended lower in hypoxemic groups (1.6 ± 1.6 and 1.1 ± 1.1 mL/kg/min) but did not reach significance.

**CONCLUSIONS:**

Maternal hyperoxia exposure is buffered by the fetoplacental circulation minimizing fetal cerebral risk. When maternal PaO_2_ < 80 mmHg, particularly < 55 mmHg, fetal carotid oxygen content declines, potentially compromising cerebral oxygen delivery.

## BACKGROUND

Pregnant women have been shown to be highly susceptible to severe respiratory disease, especially during epidemiologic surges of viral respiratory outbreaks (SARS, MERS, COVID-19, and H1N1 influenza).^1–3^ Pneumonia is the most common non-obstetric fatal complication, yet very little evidence guides current acute respiratory distress syndrome (ARDS) management in pregnancy.^4,5^ Maternal mortality from ARDS in pregnancy ranges from 14 to 39%, and perinatal mortality associated with this maternal diseases 20–30%.^6–9^ Currently, supportive care efforts in the mainstream ARDS population are aimed at lung protective tidal volume ventilation, with an ARDSnet driven protocol approach to oxygenation (sPO_2_ 88–95% and PaO_2_ 55–80 mmHg) and ventilation (pH > 7.3) to optimally mitigate risk of ventilator-induced lung injury and improve survival.^10^ However, the effect of an ARDSnet driven strategy on fetal oxygenation is unknown, limiting its clinical application and management in pregnancy. Currently, the Society for Maternal-Fetal Medicine,^11^ along with other professional obstetric societies, has recommended maintaining an oxygen saturation (sPO_2_) of at least 95% for all pregnant individuals irrespective of severity of acute respiratory illness. These recommendations are, in part, based on known maternal adaptive physiologic changes during pregnancy that include higher mean PaO_2_ at all phases of pregnancy and a known mild respiratory alkalosis shifting the oxygen-hemoglobin dissociation curve to the left and higher metabolic demand.^12–14^ However, these recommendations are not without controversy. During the height of the Delta variant outbreak with the COVID-19 epidemic, some maternal-fetal experts argued that these thresholds are possibly unrealistic to sustain in mothers with severe respiratory infections. Further, this stringent threshold may cause escalation of invasive support, while possibly providing minimal benefit to the developing fetus and potential for maternal harm.^15^ The exact maternal thresholds of acute hypoxia that are tolerable to the developing fetus, without compromising brain oxygen delivery, are unknown.

High rates of C-section deliveries and iatrogenic prematurity were reported as a result of maternal hypoxia during the COVID-19 pandemic.^16,17^ Studies to support higher sPO_2_ and PaO_2_ thresholds in pregnancy remain extremely limited. Small obstetric clinical studies have shown pregnant women exposed to subatmospheric oxygen resulted in depressed fetal activity,^18^ resulting in empiric use of maternal oxygen support during times of suspected fetal distress and routine administration during the second stage of labor. However, oxygen therapy during labor is controversial, with some studies that show an association with fetal acidosis and dysregulation in placental gas exchange, vasoconstrictive effects on umbilical and placental vessels, and generation of free radical oxygen species.^19,20^ Although classical large animal models have studied fetal responses to maternal oxygen exposure, they have primarily focused on the clinical extremes of acute hypoxia and hyperoxia.^21–24^ The direct effects of contemporary maternal oxygenation targets on fetal circulation, oxygenation, and oxygen delivery to critical organs remain unexplored, representing a key knowledge gap given the frequent clinical challenges of optimally supporting the maternal-fetal unit in pregnant patients with acute respiratory failure.

To address this gap, we compared the effects of varying maternal oxygen tension on fetal hemodynamics, fetal oxygenation, and carotid oxygen delivery in a late-preterm ovine pregnant model of acute maternal hypoxia. We specifically investigated maternal PaO_2_ targets consistent with ARDSnet guidelines (55–80 mmHg) to determine whether these levels induce fetal hypoxia and reduce carotid oxygen delivery, compared with normal maternal oxygen tension (80–150 mmHg) and at the extremes of maternal oxygenation, including hyperoxia and severe hypoxemia. We hypothesized that the maternal PaO_2_ below or within ARDSnet target range (PaO_2_ 55–80 mmHg) would cause significant fetal hypoxia and decreased carotid oxygen delivery relative to normal (PaO_2_ 80–150 mmHg) and high maternal oxygen tension parameters (PaO_2_ > 150 mmHg). In this model, maternal ewes at late preterm gestation were intubated and exposed to three levels of inspired oxygen (FIO_2_ 0.1, 0.21, and 1.0) at set intervals. Maternal and fetal blood gases, continuous pulse oximetry, and continuous fetal carotid and pulmonary flows were monitored throughout the duration of varying maternal oxygen exposure. Based on the resulting maternal PaO_2_ from these exposures, values were stratified into four target ranges for analysis: 1) PaO_2_ <55 mmHg (hypoxemia), 2) PaO_2_ 55–80 mmHg (ARDSnet recommendations), 3) PaO_2_ 81–150 mmHg (to approximate SMFM recommendations), and 4) PaO_2_ >150 mmHg (hyperoxia).

## METHODS

### Animal preparation

**Fig. 1:**
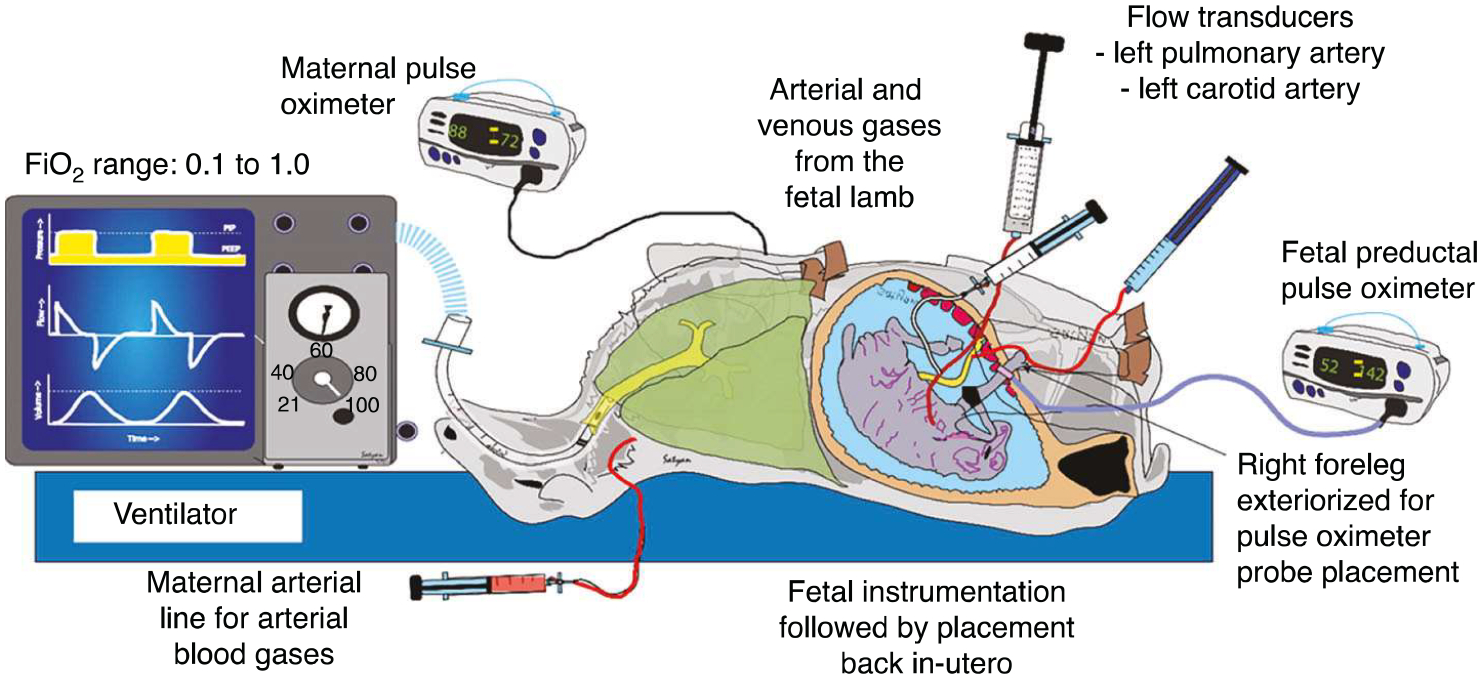
In-vivo model of late preterm pregnant ewe exposed to varying FiO_2_.

The study was approved by the Institutional Animal Care and Use Committee (IACUC) at the State University of New York, Buffalo. Time-dated, late preterm mixed-breed, Suffolk-Dorset-Katahdin breed Q fever seronegative pregnant ewes ranging from 132 to 134 days gestation (term gestation 145–147 days) were, on day of experimentation, sedated with diazepam and ketamine and subsequently intubated with a 9.5 or 10.0 mm cuffed ETT, and general anesthesia was provided by 2–3% isoflurane. Maternal tidal volume and respiratory rate were carefully adjusted by a veterinary technician on the anesthesia machine to maintain stable end-tidal CO_2_ near 40 mmHg and overall cardiopulmonary stability during general anesthesia. Ewes were continuously monitored with end-tidal CO_2_ and pulse oximetry, following previously published maternal anesthesia protocols.^25,26^ Maternal temperature was maintained near 39 °C using an electric Bair Hugger^™^ mattress. The ewes were surgically instrumented with a carotid catheter (for serial arterial blood gas analysis and continuous hemodynamic monitoring) and with an internal jugular vein catheter (for medication administration) through the experiment (Fig. 1). During this time, a hysterotomy was performed, and the in-utero fetus was partially exteriorized. The fetal lamb was intubated with a cuffed endotracheal tube and occluded to prevent exposure of the fetal lungs to atmospheric oxygen. Catheters were placed in right fetal carotid artery, right fetal jugular vein, and umbilical vein. A flow probe was placed around the left fetal carotid artery and left pulmonary artery. Following surgical instrumentation, fetus was placed back in the uterus. The uterus and abdomen were partially closed allowing access to neck catheters for access and also foreleg for fetal pulse oximetry probe placement. The maternal ewe’s FiO_2_ was randomly changed between 0.1, 0.21, and 1.0 for 20 min each, and simultaneous maternal and fetal gases were obtained at the end of each FiO_2_ exposure. Hemoglobin levels were measured on all blood gases, and estimated cerebral oxygen delivery of the fetus in-utero, inferred from the continuous fetal carotid flow measurement data, was calculated. Continuous pulse oximetry measurements of both maternal ewe and fetus-in-utero (preductal—tongue or right forelimb) were obtained throughout the duration of the study.

### Data calculation and statistical analysis

A formal power analysis was conducted using preliminary fetal lamb data, which indicated a baseline fetal PaO_2_ of 20 ± 4 mmHg, consistent with published reports.^27,28^ Based on Konduri et al. a clinically meaningful change of approximately 7 ± 2 mmHg in fetal PaO_2_ produces substantial alterations in pulmonary vascular resistance and blood flow; we therefore selected an effect size of 5 mmHg. Assuming a two-sided α of 0.05, 80% power (*β* = 0.2), and a standard deviation of 4 mmHg, the required sample size was 25 animals, with approximately 12 per group for pairwise comparisons. Analysis used parametric statistics obtained with IBM SPSS Statistics (Version 27), and comparisons of data were made between 4 stratified maternal PaO_2_ groups (<55 mmHg, 55–80 mmHg, 81–150 mmHg, and > 150 mmHg) as mentioned previously. Significant differences were reported with a p-value of < 0.05. Results were compared using ANOVA statistical test and post-hoc pairwise comparisons with Bonferroni method. Linear regression models were also used, as appropriate, to evaluate the relationship between continuous variables.

Carotid fetal oxygen delivery and extraction were calculated using calculations of fetal oxygen content of the arterial (CaO_2_) and venous (CvO_2_) blood. Formula for oxygen content was: (Hb*1.34*SaO_2_) + (PaO_2_*0.0031) in mL/dL. The CvO_2_ was subtracted from the CaO_2_ to calculate arteriovenous oxygen difference. Fetal arterial saturation and PaO_2_ were obtained from fetal carotid blood gas measurements, with the venous oxygen content calculated from the venous blood gas measured from the right fetal jugular bulb. This enabled us to infer estimated cerebral oxygen extraction. “Cerebral” oxygen delivery was inferred from the product of carotid flow and arterial oxygen content.

## RESULTS

A total of 31 maternal ewes were included in the analysis. Table 1 shows baseline characteristics, and Table 2 shows the 4 distinct stratified maternal PaO_2_ categories achieved with variable FiO_2_ levels throughout the duration of the study. Available maternal and fetal blood gas data are presented in the online supplement, stratified by maternal PaO_2_ category (Supplemental Table S1). No significant differences were observed across maternal PaO_2_ groups with respect to maternal pH or lactate in this subgroup of ewes; all values remained within physiologic ranges with minimal variability.

### Maternal PaO_2_ categories and fetal oxygenation

**Fig. 2:**
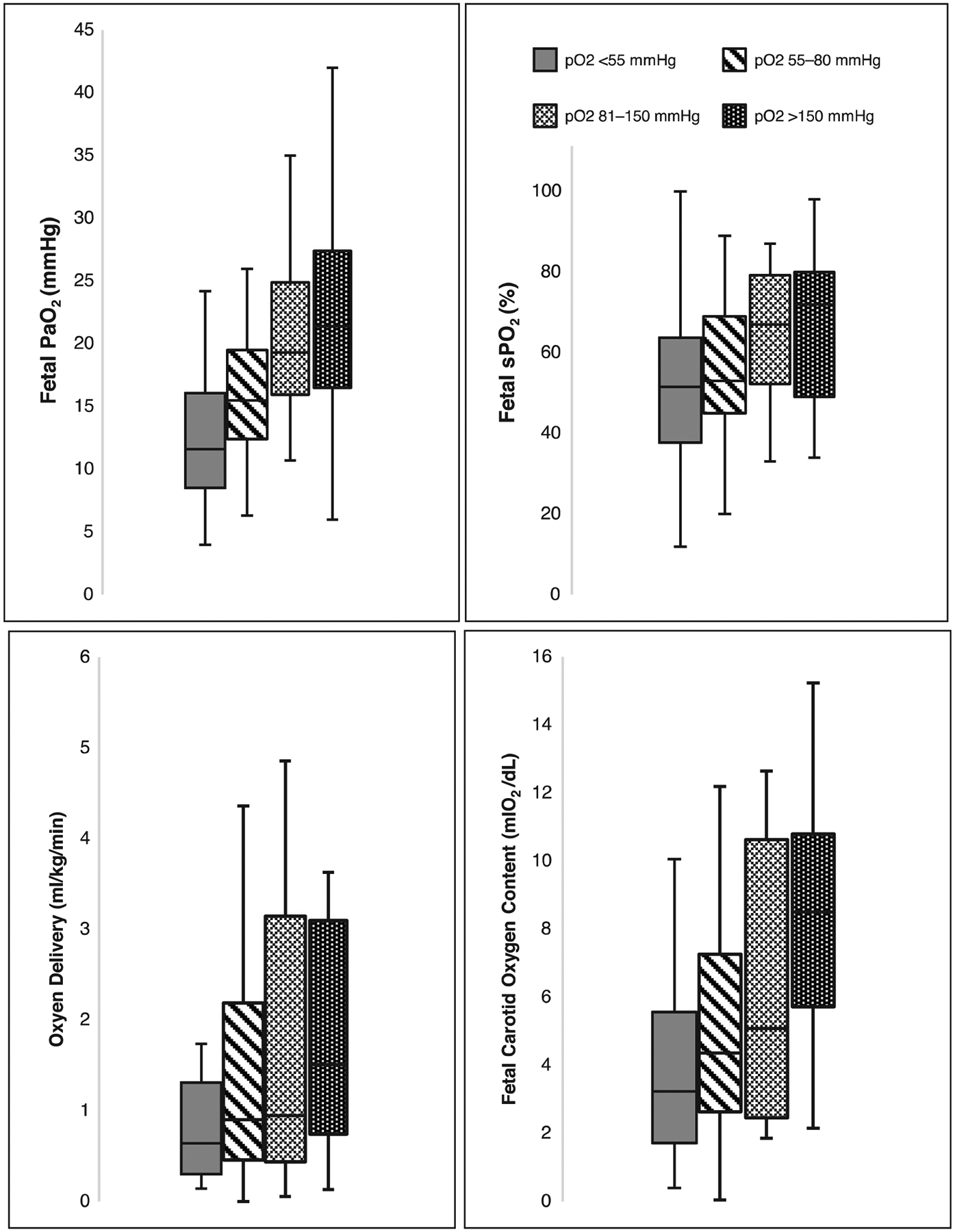
Distribution of fetal oxygen values (tension, pulse oximetry, delivery, content) and fetal pulmonary blood flow across four groups of maternal oxygen tension (<55, 55–80, 81–150, >150).

**Fig. 3:**
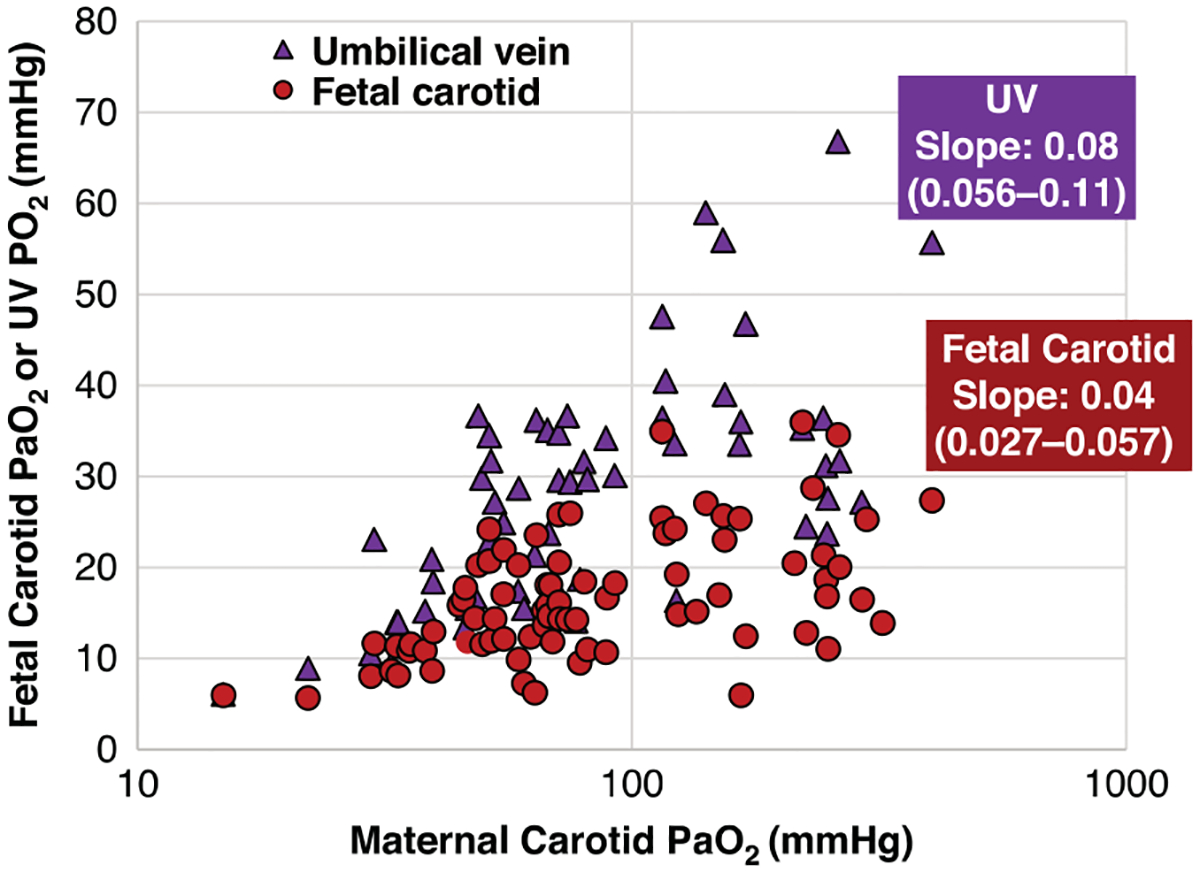
Relationship between maternal carotid oxygen tension and fetal oxygenation. Between maternal values of 80 and 150 mmHg fetal arterial oxygen tension (PaO_2_) remains relatively constant suggestive of fetal autoregulation at this given range of maternal arterial oxygen tensions.

A decrease in maternal PaO_2_ levels resulted in significantly lower fetal PaO_2_ levels (*p* < 0.001, Table 2). The difference fetal sPO_2_ levels did not reach statistical significance (*p* = 0.1, Table 2 and Fig. 2). In the post-hoc pairwise comparisons, a significant decrease in fetal PaO_2_ was observed when comparing maternal PaO_2_ < 55 mmHg to normal maternal oxygen tension (PaO_2_ 81–150 mmHg). There was no significant difference in fetal PaO_2_ between the ARDSnet (PaO_2_ 55–80 mmHg) and SMFM (PaO_2_ 81–150 mmHg) recommended maternal oxygen tension range. However, an ARDSnet-driven maternal oxygenation strategy did result in levels that could threaten adequate fetal oxygenation with mean fetal carotid PaO_2_ of 16.1 ± 5.1 mmHg and umbilical venous PvO_2_ of 27.1 ± 6.6 (Table 2, Figs. 2 and 3). Further, the percentage of fetal lambs that were acutely hypoxic (umbilical venous PvO_2_ < 30 mmHg) were 85% and 77% among the maternal PaO_2_ groups of < 55 mmHg and ARDSnet parameters of 55–81 mmHg, compared to 27% and 25% of lambs in the maternal PaO_2_ groups of 81–150 and >150. Between maternal arterial oxygen tension ranges of 81–150 mmHg, fetal arterial oxygen tension (PaO_2_) remained relatively constant, suggestive of fetal autoregulation within this given maternal range (Fig. 3).

### Maternal PaO_2_ categories and fetal hemodynamics and carotid blood flow

#### Fetal hypoxia and mean blood pressure.

There was no observed or significant change to mean or fetal systolic blood pressure stratified by maternal oxygen tension groups (Table 2).

#### Fetal hypoxia and carotid blood flow.

**Fig. 4:**
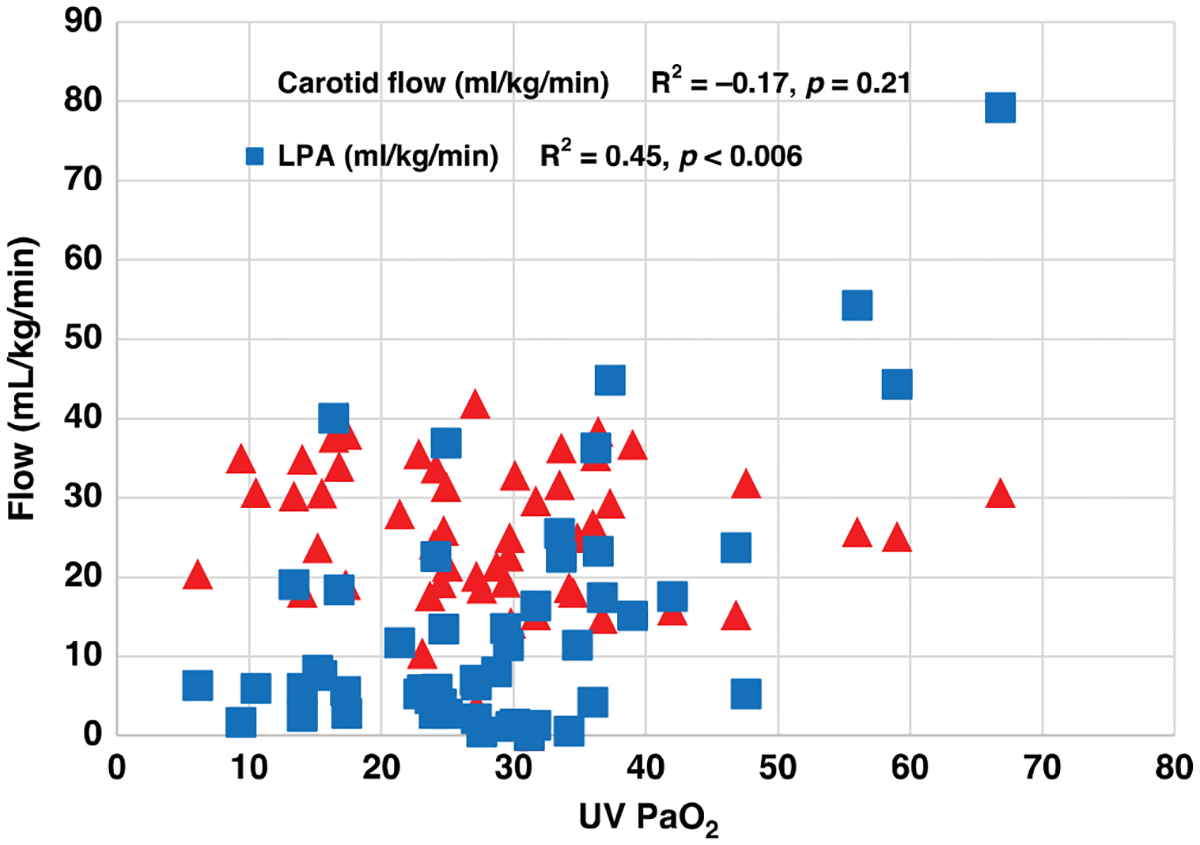
Relationship between fetal oxygenation defined as umbilical venous PaO_2_ and fetal carotid blood flow and left pulmonary arterial flow in-utero. Pulmonary blood flow (blue) and carotid blood flow (red). Pulmonary blood flow increases with increasing oxygen tension returning from the umbilical vein. Despite this, carotid blood flow remains constant.

There was no significant change in fetal carotid flow over a wide range of fetal and maternal oxygen tensions, with no observed compensatory increase to fetal carotid brain blood flow at levels of fetal hypoxia (Fig. 4). As fetal oxygen tension levels increased to normal to high levels, cerebral blood flow stayed constant with also no significant changes (*r*^2^ = −0.17, *p* = 0.21, Fig. 4).

#### Fetal hypoxia and pulmonary blood flow.

There was a significant moderate positive correlation between pulmonary blood flow as maternal oxygen tension increased (*R*^2^ = 0.45, *p* < 0.001, Fig. 4).

### Maternal PaO_2_ categories and fetal carotid oxygen delivery

There was a decrease in fetal cerebral oxygen delivery in the low PaO_2_ maternal subgroup of < 55 mmHg (1.08 ± 1.1 ml/min) when compared to fetal brain oxygen delivery at normal maternal oxygen tensions (2.1 ± 1.7 ml/min). However, this pairwise comparison was not statistically significant (*p* = 0.33). Fetal cerebral (carotid) oxygen delivery remained nearly constant among normal maternal oxygen tensions of 81–150 mmHg compared to high supraphysiologic maternal oxygen tensions of PaO_2_ > 150 mmHg (2.1 ± 1.7 vs 2.0 ± 1.2, Table 2). At ARDSnet threshold (maternal PaO_2_ 81–150 mmHg) and at maternal PaO_2_ thresholds < 55 mmHg, we observed a stepwise decline in mean fetal cerebral oxygen delivery (1.64 ± 1.6 and 1.08 ± 1.1 respectively) but ultimately there was no significant differences among the group means (Table 2 and Fig. 2). The percentage lambs with fetal cerebral oxygen delivery less than the 50th percentile from baseline were 76.2% and 71.4% respectively in the maternal PaO_2_ groups of < 55 and ARDSnet 55–80 compared to the 45.5% and 43.8% of the lambs in the maternal PaO_2_ groups of 81–150 and > 150 mmHg.

## DISCUSSION

Given higher susceptibility of pregnant women to severe respiratory illness and limited data supporting the exact maternal oxygenation thresholds that can adeqeuately support fetal oxygenation, we sought to determine the relationship between varying maternal PaO_2_ on fetal circulation and oxygenation in-utero in a large mammalian preclinical in-vivo model of acute maternal hypoxia. Most pertinently, we sought to determine if application of ARDSnet oxygenation (PaO_2_ 55–80 mmHg) threshold used in mainstream ARDS population is applicable and safe in pregnancy. We oxygenated late preterm pregnant sheep at varying FiO_2_ levels to induce acute maternal hypoxia and observed that at an ARDSnet driven oxygenation parameters, there were significant declines in fetal carotid PaO_2_ and umbilical venous PvO_2_ compared to maternal ewes oxygenated to >150 mmHg. At maternal PaO_2_ <55 mmHg, fetal carotid PaO_2_ and umbilical venous PvO_2_ were significantly reduced compared with both >150 mmHg and normal oxygenation (81–150 mmHg) groups. Although fetal cerebral oxygen delivery trended downward across decreasing maternal oxygenation groups, these differences were not statistically significant, despite significant reductions in fetal carotid oxygen content at PaO_2_ ≤80 mmHg when compared with the >150 mmHg group. There were no observed differences in fetal oxygenation parameters and fetal carotid oxygen delivery in maternal ewes that were oxygenated to normal maternal PaO_2_ parameters versus at supra-physiologic levels of maternal PaO_2_, suggesting that the fetoplacental circulation is fairly effective in buffering against maternal hyperoxia.^29^ Further, lower maternal PaO_2_ targets correlated with an observed trend toward declines in fetal pulmonary blood flow, with no associated changes to fetal carotid blood flow.

Our findings support the historical consensus that the developing fetus can thrive under a relative hypoxic environment, and the placenta serves as a physiologic buffer in reducing oxygen exposure to the developing fetus.^30^ High maternal PaO_2_ of >150 mmHg and at 81–150 mmHg result in similar fetal umbilical venous PvO_2_, consistent with historical normative parameters (PvO_2_ 30–34 mmHg and oxygen saturations of 67–75% at term gestation), and fetal carotid oxygen delivery remained constant across these two groups. At low baseline partial pressure of fetal venous oxygen, we speculate that fetal oxygen delivery is supported by fetal adaptations that include high fetal cardiac output, high fetal hemoglobin concentration, and the presence of various fetal circulatory shunts, in particular the relative volume of shunting across the foramen ovale.^29^ Within the ARDSnet PaO_2_ target range (55–80 mmHg), fetal oxygenation trended downward, with significant reductions compared with the maternal hyperoxia group and evidence of absolute hypoxemia (mean PvO_2_ 27.1 ± 6.6 mmHg). However, no significant differences were observed between ARDSnet and SMFM maternal oxygenation targets across measures of fetal oxygenation. At maternal PaO_2_ less than 55 mmHg, fetal oxygenation declined significantly compared with normal maternal oxygenation groups. Although fetal carotid oxygen delivery decreased progressively with declining maternal PaO_2_, these reductions did not reach statistical significance across groups.

During the height of the delta variant surge during the COVID-19 pandemic, Eid et al. called for a re-appraisal of maternal oxygenation targets with acute respiratory disease, advocating for 92–96% goal as long as fetal status remains reassuring.^15^ The World Health Organization also suggested maintaining sPO_2_ at 92–96% in pregnancy individuals with severe respiratory infections secondary to COVID-19. This recommendation was based on expert opinion suggesting a PaO_2_ of 60–70 mm Hg as a reasonable maternal target that would maintain placental oxygen gradient and adequate fetal oxygen delivery, in part driven by reassuring perinatal outcomes of pregnant women who live under high altitude (where the partial pressure of oxygen results in PaO_2_ levels of ~60 mmHg). However, chronic exposure to subatmospheric oxygen allows for maternal compensatory mechanisms that include relative maternal polycythemia, that aid in preserving maternal oxygen delivery and do not mimic the conditions of acute maternal hypoxia. Higher maternal oxygenation targets advocated by Society of Maternal Fetal Medicine are based on historical higher normative maternal PaO_2_ in pregnant persons (at all phases of pregnancy) and adaptive respiratory changes that optimize fetal oxygenation.^12–14^ However, there is limited data to support these advocated targets and there is general clinical equipoise on what goal lower and upper maternal oxygen targets needs are in pregnant women, with further uncertainty regarding ideal oxygenation targets in pregnant persons with acute parenchymal lung disease to best support maternal-fetal wellbeing.

Our findings do support that maintaining a maternal oxygenation threshold of 95–99% best supports fetal carotid oxygen content and possibly fetal oxygenation, and there is likely no additional benefit to fetal cerebral oxygen delivery with additional oxygen support above these parameters. Fetal circulation in-utero and the placenta act as an important physiologic buffer to high maternal oxygen content and regulates oxygen exposure and delivery to the brain over a narrow range. Lowered maternal PaO_2_ thresholds of 55–80 mmHg can lead to low fetal carotid oxygen content and possibly fetal hypoxia and may still jeopardize adequate oxygen delivery to vital organs. Further studies are needed to distinguish whether these maternal PaO_2_ thresholds of 55–80 mmHg truly compromise vital fetal organ development.

Fetal circulation in-utero has the adaptive ability to preferentially shunt higher oxygenated blood to critical organs such as heart and brain.^31^ Fetal circulation runs in a parallel series. The right-sided cardiac output enters the descending aorta through the patent ductus arteriosus to supply blood to the lower side of the body. The left-sided cardiac output preferentially provides higher oxygenated blood to cerebral and coronary circulation. Oxygenated umbilical venous return enters the right atrium and selectively shunts from the right to left side of the heart through the foramen ovale, while under normal fetal conditions of high pulmonary vasoconstriction and limited pulmonary blood flow.^29^ The balance of blood shunted through the foramen ovale and ductus arteriosus is finely balanced in-utero, best maintaining cerebral oxygen delivery within a constant narrow range despite extremes of maternal oxygen exposure. At a maternal PaO_2_ > 150 mmHg, we observed only marginal increases in fetal right atrial PaO_2_ and umbilical vein PvO_2_, mainly due to the reduced oxygen uptake and diversion of blood from the placenta. Further, high maternal oxygenation > 150 mmHg also results in reduced PVR and higher pulmonary blood flow, increasing deoxygenated blood returning to the left atrium and further buffering the oxygen content of the left ventricle and carotid artery, maintaining carotid oxygen delivery at a constant rate. In contrast, at lower maternal PaO_2_ levels, a decrease in pulmonary blood flow and a significantly lower umbilical venous to fetal carotid PaO_2_ gradient was observed, indicating preferential flow of oxygenated blood to the left ventricular output to maintain cerebral oxygenation. Despite this buffering mechanism, at a maternal oxygen PaO_2_ threshold of <55 mmHg, fetal carotid oxygen tension significantly declines. Further, at these levels, there was no observed compensatory increase in carotid blood flows, further threatening adequate cerebral oxygen delivery. These results suggest that slightly higher maternal PaO_2_ and sPO_2_ levels as recommended by SMFM may result in optimal fetal cerebral oxygen delivery, while fetoplacental circulatory shunts buffer and limit fetal hyperoxia. Strengths of this study include the unique aspect of a large mammalian in-vivo model capable of fetal instrumentation in-utero that allows assessment of maternal-fetal interactions in real-time. This is the first large mammalian model to describe the role of maternal oxygen content across a wide spectrum of relevant physiological ranges and its impact on fetal oxygenation, pulmonary vascular resistance, carotid blood flow, and regulation of cerebral brain oxygen delivery. Although classic large animal translational studies have investigated hyperoxia and hypoxia exposure in the past, none have examined these effects in the context of today’s standards of maternal oxygenation targets.^21–24^ Clinical trials remain limited in the obstetric population due to unknown fetal risk to various interventions. Current recommendations are driven largely by expert opinion with general equipoise on the clinical management of pregnant women with acute respiratory failure. Clinical translational models that allow for fetal instrumentation in-utero, enable for the comprehensive study of critical maternal-fetal interactions with maternal oxygen titration manipulation. This model allows for a more comprehensive understanding of fetal physiology with maternal oxygen exposure that cannot be carried out in pure clinical studies.

There are limitations of this study that impact interpretation of these findings. The study investigated critical maternal-fetal interactions in the setting of reduced FiO_2_ and not hypoxemia secondary to acute lung disease that results from intrapulmonary shunt, ventilation-perfusion mismatch and reduced diffusion capacity. The direct effect on maternal-fetal interactions from lower maternal oxygenation thresholds from alveolar disease and systemic inflammation (ie. ARDS) needs further investigation. The short temporal nature of reducing maternal FiO_2_ is intrinsically limited in its evaluation, and we may not have fully observed possible adaptive and dynamic changes to fetal circulation over a longer period of time of varying maternal oxygen exposure. Due to the cross-over nature of the study design, outcomes are limited to fetal oxygenation parameters and circulation in-utero. Further, the threshold and duration of reduced carotid oxygen delivery required to cause hypoxic neurologic injury remains unknown, highlighting the need for further investigation to contextualize our findings clinically.

Additional limitations pertain to the physiological measurements and the methodology employed in this study. Although we used a well-established laboratory anesthesia protocol,^25,26^ it did not strictly follow a clinical anesthesia approach^32^ which may confound maternal and fetal physiological assessments. Fetal carotid flow was measured unilaterally and used as a surrogate for cerebral blood flow, as in prior studies.^33,34^ However, carotid flow may not fully reflect true cerebral perfusion due to extracerebral contributions and the assessment of a single carotid artery, which may partially account for the absence of significant changes observed. Further, maternal carotid flow was not directly measured, limiting further evaluation of the maternal-fetal oxygen delivery relationship. Finally, we did not evaluate oxidative stress markers in the fetal brain and maternal lung.

## CONCLUSION

In conclusion, in a preclinical in-vivo late-preterm ovine pregnancy model of varying oxygen exposure, maternal PaO_2_ < 55 mmHg caused significant declines in fetal oxygenation compared to normal maternal oxygenation (SMFM current recommendations), while ARDSnet oxygenation goals also reduced fetal oxygenation and content relative to maternal hyperoxia. Despite these reductions, fetal carotid oxygen delivery did not significantly differ across the groups. High or normal maternal oxygen tensions were buffered by the placenta and fetal circulation, maintaining stable carotid oxygen delivery and minimizing risk of oxidative stress. Pulmonary vascular resistance may be dynamic in the human fetus and is an important regulator of distribution of cardiac output and oxygen delivery to the brain and heart, modulating oxygen delivery over a narrow and constant range. Further studies are needed to determine what the exact maternal PaO_2_ thresholds that threaten fetal oxygen delivery to vital organs with women with acute respiratory disease. Our results suggest that in women with acute hypoxia from low inspired oxygen content, maternal oxygen ranges within ARDSnet parameters (PaO_2_ of 55–80 mmHg, sPO_2_ 88–95%) may compromise fetal oxygenation, while the effects on cerebral oxygen delivery remain uncertain and potentially at risk, warranting further study of its safety in pregnancy.

## Supplementary Material

supplement

**Supplementary information** The online version contains supplementary material available at https://doi.org/10.1038/s41390-026-04858-z.

## Figures and Tables

**Table 1.** Baseline characteristics of maternal ewe and fetal lamb prior to maternal oxygenation strategy

Baseline characteristics (mean ± SD)	*n* = 31
**Maternal baseline**	
Maternal arterial PaO_2_ (mmHg)	89.2 ± 52.2
Maternal alveolar PAO_2_ (mmHg)	102.3 ± 10.5
Multiple gestation n (%)	12 (39%)
Maternal Arterial to Umbilical Venous PO_2_ gradient (mmHg)	61.9 ± 54.0
**Fetal baseline**	
Fetal lamb weight (kg)	3.62 ± 0.89
Fetal systolic blood pressure (mmHg)	51 ± 9
Fetal diastolic blood pressure (mmHg)	37 ± 10
Fetal mean arterial pressure (mmHg)	43 ± 11
Umbilical venous PO_2_ (mmHg)	31.1 ± 9.0
Fetal right carotid PaO_2_ (mmHg)	18.9 ± 7.3
Umbilical venous to fetal right carotid PaO_2_ gradient (mmHg)	11.5 ± 7.1
Right fetal right atrial PaO_2_ (mmHg)	15.0 ± 6.0
Fetal carotid oxygen content (mL/dL)	5.8 ± 3.7
Fetal “cerebral” oxygen delivery (mL/kg/min)	1.48 ± 1.30

**Table 2.** Oxygen tension and blood flows in catheterized maternal and fetal vessels stratified by maternal oxygen tension levels (PaO_2_-mmHg)

Mean ± SD	PaO_2_ < 55 *N* = 26	PaO_2_ 55–80 *N* = 28	PaO_2_ 81–150 *N* = 17	PaO_2_ > 150 *N* = 23	*p*-value
**Maternal oxygenation and hemodynamics**					
Maternal arterial PaO_2_ (mmHg)	39.7 ± 12.0^b,c,d^	66.6 ± 6.8^b,e,f^	108.3 ± 22.3^c,e,g^	245 ± 62.0^d,f,g^	**<0.001**
Maternal alveolar PAO_2_ (mmHg)	40.4 ± 36.8^b,c,d^	197 ± 225^e,b,f^	400 ± 289^c,e^	544 ± 239^d,f^	**<0.001**
Maternal End-Tidal CO_2_ (mmHg)	38.6 ± 5.4	39.6 ± 5.6	38.7 ± 6.5	38.4 ± 4.5	0.83
Maternal Heart Rate (bpm)	113 ± 29	96 ± 14	112 ± 26	102 ± 18	0.05
**Fetal oxygenation and circulation**					
Fetal systolic BP (mmHg)	53 ± 16	53 ± 9	52 ± 9	54 ± 14	0.921
Fetal mean BP (mmHg)	39 ± 14	44 ± 12	43 ± 11	44 ± 15	0.574
Umbilical venous PvO_2_	20.1 ± 8.9^c,d^	27.1 ± 6.6^f^	35.4 ± 11.3^c^	38.3 ± 12.3^d,f^	**<0.001**
Maternal arterial to Umbilical venous PO_2_ gradient	27.4 ± 10^c,d^	51.0 ± 7.8^f^	88.4 ± 22.8^c,g^	221.9 ± 62.4^d,f,g^	**<0.001**
Fetal right carotid PaO_2_	12.3 ± 5.0^c,d^	16.1 ± 5.1^f^	20.6 ± 6.6^c^	22.6 ± 9.0^d,f^	**<0.001**
Fetal preductal sPO_2_	51 ± 20	55 ± 16	65 ± 16	66 ± 19	0.11
Umbilical venous to fetal carotid PO_2_ gradient	7.6 ± 7.3^d^	10.1 ± 7.1	13.0 ± 9.6	16.7 ± 10.9	**<0.05**
Fetal right atrial PaO_2_^a^	9.4 ± 3.9^c,d^	14.1 ± 4.6^f^	15.7 ± 6.5^c^	20.9 ± 11.8^d,f^	**<0.001**
Fetal pulmonary blood flow (ml/kg/min)	7.2 ± 7.7	10.7 ± 9.6	14.8 ± 17.1	18.0 ± 19.6	0.075
Fetal carotid blood flow (ml/kg/min)	22.9 ± 8.8	24.8 ± 9.4	24.8 ± 10.7	22.8 ± 8.6	0.87
Fetal carotid Oxygen Content (ml/dL)	4.03 ± 3.07^d^	5.18 ± 3.70^f^	6.65 ± 4.29	8.16 ± 3.32^d,f^	**<0.05**
Fetal cerebral oxygen delivery (ml/kg/min)	1.08 ± 1.1	1.64 ± 1.6	2.1 ± 1.7	2.0 ± 1.2	0.89

a Right atrial blood gases were drawn from a jugular venous line advanced into the right atrium.

b *p*-value < 0.05, with pair-wise comparison of < 55 vs 55–80 mmHg maternal PaO_2_ group.

c *p*-value < 0.05, with pair-wise comparison of < 55 vs 81–150 mmHg maternal PaO_2_ group.

d *p*-value < 0.05, with pair-wise comparison of < 55 vs >150 mmHg maternal PaO_2_ group.

e *p*-value < 0.05, with pair-wise comparison of 55–80 vs 81–150 mmHg maternal PaO_2_ group.

f *p*-value < 0.05, with pair-wise comparison of 55–80 vs >150 mmHg maternal PaO_2_ group.

g *p*-value < 0.05, with pair-wise comparison of 81–150 vs >150 mmHg maternal PaO_2_ group.

## Data Availability

The datasets generated during and/or analyzed during the current study are available from the corresponding author on reasonable request.

## IMPACT:

- Pregnant women are highly susceptible to severe respiratory illness and ARDS, yet guidance on optimal maternal oxygen targets is limited.
- We investigated how varying maternal PaO_2_ levels affect fetoplacental circulation and in-utero oxygenation.
- Using a large mammalian model of acute maternal hypoxia, we assessed whether maternal ARDSnet oxygenation targets (PaO_2_ 55–80 mmHg, SpO_2_ 88–95%) can be safely applied in pregnancy. Fetal carotid oxygen content decreased at maternal ARDSnet targets and when below PaO_2_ 55 mmHg, with a non-significant trend toward reduced fetal cerebral oxygen delivery. Application of a maternal ARDSnet strategy may lead to suboptimal fetal oxygenation.
