# Supplementary material for: Impact of varying range of maternal oxygenation targets on fetal oxygenation and fetoplacental circulation in an ovine model of pregnancy
Source: Pediatr Res. Author manuscript; Available in PMC 2026 May 2. (PMC13135292; doi:10.1038/s41390-026-04858-z)
Supplement: supplement [file NIHMS2168153-supplement-supplement.pdf]

Title: Impact of Varying Range of Maternal Oxygenation Targets on Fetal Oxygenation and Fetoplacental Circulation in an Ovine Model of Pregnancy

e-Supplement

Supplemental Table S1.

Mean ± SD	PaO_2 <55 N= 6	PaO_2 55-80 N=10	PaO_2 81-150 N=3	PaO_2 >150 N=7	p-value
**Maternal Oxygenation and Hemodynamics in Subgroup of Maternal Ewes**					
Maternal pH	7.4±0.08	7.37±0.05	7.42±0.04	7.36±0.06	0.05
Maternal Lactate (mmol/L)	1.2±0.24	1±0.24	1±0.1	1.14±0.23	0.34
Fetal pH	7.02±0.09	7.06±0.06	7.07±0.1	7.1±0.04	0.19
